# Antibacterial properties and GC-MS analysis of ethyl acetate extracts of *Xerophyta spekei* (Baker) and *Grewia tembensis* (Fresen)

**DOI:** 10.1016/j.heliyon.2023.e14461

**Published:** 2023-03-11

**Authors:** Paul Ochieng Nyalo, George Isanda Omwenga, Mathew Piero Ngugi

**Affiliations:** a Department of Biochemistry, Microbiology and Biotechnology, Kenyatta University, P.O Box 43844-00100, Nairobi, Kenya; b Penda Health (K) Ltd, Medical Laboratory Department, P.O Box 22647-00100, Nairobi, Kenya

**Keywords:** Antibacterials, *Grewia tembensis*, *Xerophyta spekei*, Infectious diseases, Ethyl acetate

## Abstract

Conventional antibiotics are associated with various side-effects. Therefore, there is need of using plant-derived antibiotics with fewer side-effects. *Grewia tembensis* and *Xerophyta spekei*, which have been extensively utilized in the Mbeere community, were studied to support their folkloric use and demonstrate their antibacterial capabilities. *Salmonella* Typhi ATCC 1408, *Bacillus subtilis* ATCC 21332, *Staphylococcus aureus* ATCC 25923, and *Escherichia coli* ATCC 25922 were all used in this study. As a standard reference, Ciprofloxacin (100 μg/ml) was employed, and 5% DMSO was used as a negative reference. Tests for antibacterial activities included disc diffusion, minimum inhibitory concentrations, and bactericidal concentrations. *G. tembensis* exhibited effects on *S. aureus* only with Mean Zone Inhibition (MZI) of 07.07 ± 0.07 to 12.33 ± 0.33 mm and 08.33 ± 0.33 to 11.67 ± 0.33 mm for stem bark and leaf extracts respectively. While *X. spekei* extract had effects on *S. aureus* with MZI of 07.67 ± 0.33 to 14.67 ± 0.33 mm and *B. subtilis* with MZI of 09.67 ± 0.33 to 14.33 ± 0.33 mm. Ciprofloxacin demonstrated significantly higher activities as compared to the plant extracts in all the concentrations (*p* < 0*.*05), while 5% DMSO had no activity. GC-MS analysis demonstrated the availability of compounds with known antibacterial effects. Therefore, the current study recommends ethnomedicinal and therapeutic use of *G. tembensis* and *X. spekei* as antibacterial agents.

## 1 Introduction

The spread of infectious diseases has reportedly been facilitated by increased human population in major cities, environmental degradation, and lack of proper health care services [1]. Some conditions that lead to the increased number of individuals with compromised immunity have also led to a large number of people being subjected to bacterial infections [2]. There has been a rise in the use of counterfeit antibacterial agents due to the increased expense of medication, especially in underdeveloped countries, some of which are of sub-standard quality. Their use has led to a loss of lives and resulted in antibacterial drug-resistant pathogens [3].

Antibacterial resistance to commonly used antibiotics is currently increasing at an alarming rate, which has complicated the management of bacterial infections [4]. Some of the elements that cause this phenomenon include misuse of over-the-counter antibiotics, noncompliance to antibiotic dosage due to their adverse side effects, and use of under-dose due to the high expense of medication [4,5]. Therefore, there is a critical need for alternative and complementary antibacterial therapies which are affordable and have fewer side effects.

Since time immemorial, medicinal plants have been used as therapeutic drugs to prevent or heal diseases as they are associated with fewer side effects and are affordable [6]. Natural antibacterial components have been discovered in medicinal plants. However, only a few have been scientifically evaluated and validated [7]. The two plants used in the current study; *Xerophyta spekei* and *Grewia tembensis* are used by herbalists as medicinal herbs in the Mbeere community in Embu County [8]. However, their ethyl acetate antibacterial activities have yet to be shown scientifically. Therefore, this experiment was executed to explore, establish and confirm the two medicinal plant extracts’ antibacterial capabilities *in vitro*.

*X. spekei,* also called *Vellozia spekei*, is a member of the Velloziaceae family. It's common in Kenya, Zambia, Tanzania, Zimbabwe, and Ethiopia. It is a shrub of about 2–5 m tall with 6–12 cm thick branched stems. The leaves are congregated on one edge of the branch [9]. The flowers appear 1–4 in a leaf axial at the stem apex [9]. In the Embu community, it is known as Kianduri and is used to treat dog bites and diabetes [8]. The Mbeere community uses it to treat dog bites and diabetes [8]. Traditional herbalists in South Africa use it as an analgesic and an anti-inflammatory [10].

*G. tembensis* is a small multi-stemmed perennial shrub about 4 m high with long, narrow, smooth, gray stems belonging to the family Tiliaceae. The leaves are green, thinly hairy, and slightly rough above with jagged margins. Flowers are white to pink, and the fruits are usually 2–4 lobed, green when young but turn orange when mature. It grows in moderately dry areas in Eastern Africa [11]. The Kipsigis call it Chesarebut, the Mbeere call it Muruba [12]. *G. tembensis* is used in Djibouti to treat microbial infections like abscesses and furuncle [13]. The Turkana community uses *G. tembensis* to treat coughs and eat its fruits as food [14]. The Kamba community calls it Muvindaviti or Mutuva and uses its roots to treat typhoid [12].

Ethyl acetate was chosen as the extraction solvent in the current study because it has been demonstrated to be a medium polar solvent with the capacity to extract molecules with less polarity as well as slightly polar compounds [15]. Prior research has also demonstrated that ethyl acetate-based extraction provided excellent secondary metabolite yields, which in turn led to excellent antibacterial outcomes [15]. According to past literature, use of ethyl acetate solvent extraction has shown better antibacterial efficience in comparison to other organic solvents [16,17].

## 2 Materials and methods

### 2.1 Plant materials collection and preparation

In May 2021, a practicing village traditional herbalist assisted in the collection of fresh *X. spekei* (whole plant without roots) and *G. tembensis* (leaves and stem barks) parts from Gikuyari village in Embu County, Kenya. The plants were brought to the university, where they were confirmed by a qualified taxonomist and a sample of each plant was deposited in the Kenya National Museum (KNM) herbarium for future reference. For *X. spekei* and *G. tembensis*, respectively, voucher numbers for the specimens were assigned as PN/002/27698/2018 and PN/003/27698/2018. The plants were cleaned thoroughly with running tap water, rinsed with distilled water (DH2O), and then cut into little pieces. Following a 28-day period of shade drying, the plants were ground into fine powder and stored at room temperature in tightly sealed containers awaiting the extraction process.

### 2.2 Extraction procedure

*G. tembensis* stem bark (400 g) powder was soaked in 1200 ml of ethyl acetate solvent. In addition, 800 g of *X. spekei* whole plant dry powder and 300 g of *G. tembensis* dry leaves powder were separately soaked in 2.4 L and 0.9 L of the solvent, respectively. All the plants were soaked for 72 h. To guarantee complete dissolution, the solutions were occasionally whirled. The solutions were then decanted and vacuum filtered using Whatman's filter paper No. 1 in a Buchner funnel. The filtrates were thereafter separately concentrated with the help of a rotary evaporator at 90 rpm at 60 °C under vacuum to evaporate the solvent. The resultant extracts were stored at 4 °C in clean, sterile glasses awaiting the bioassay experiments.

### 2.3 Experimental design

This particular study was carried out using a completely randomized study design.

### 2.4 Bacterial test organisms and controls

*B. subtilis* ATCC 21332, *S. aureus* ATCC 25923, *E. coli* ATCC 25922, as well as and *S.* Typhi ATCC 1408 bacterial isolates were obtained from Kenyatta University's Microbiology Laboratory. Ciprofloxacin was used as a positive reference antibiotic, while 5% DMSO was used as a negative reference.

#### 2.4.1 Maintenance of bacterial stock cultures

To get fresh colonies, the stock bacterial pathogens were streaked on Mueller Hinton Agar followed by 24-h incubation at 37 *°*C. To obtain fresh bacterial growth suspensions, a sterile wire loop was used to pick three to four colonies and mix with 10 ml of sterile Mueller Hinton Broth in sterile glass tubes, the tubes were then incubated at 37*°*C for 24-h. The freshly obtained bacterial suspensions were maintained at 4*°*C [18].

### 2.5 Sterile paper discs preparation

Whatman's filter papers No 1 were punched with the aid of a paper punch to prepare 6 mm diameter paper discs. Prior to sterilization, they were placed in bijou bottles and autoclaved at 121 °C for 15 min. After which, they were stored in a dry clean place until use.

### 2.6 Preparation of extracts dilutions and impregnation of discs

One hundred mg of *G. tembensis* and *X. spekei* extracts were weighed then placed in sterile 2 ml micro-centrifuge tubes. A 100 mg/ml stock concentration was prepared by adding 1 ml of 5% DMSO to the weighed extracts, the mixture was then properly vortexed followed by sonication to enable complete dissolution. Concentrations from 3.125 mg/ml to 50 mg/ml were prepared by serial dilutions which were made by mixing 500 μL of the extracts’ stock solution with 500 μL of 5% DMSO. Fifteen μL of the serially diluted extracts were used to impregnate sterile paper discs. Prior to being place on the surface of the inoculated media, the impregnated discs were left to air dry for about 20 min in a biosafety cabinet. As a positive antibiotic reference, 100 μg of ciprofloxacin powder dissolved in 1000 μL of sterile normal saline was used, while 5% DMSO was used as a negative reference.

### 2.7 Antibacterial activity test

Disc diffusion technique done in triplicates as previously explained by Hudzicki [19] was used to determine the extracts antibacterial effectiveness. Using sterile cotton swabs, bacterial inocula were evenly streaked on the surface of already prepared Mueller Hinton Agar media. After which, the inoculated culture plates were left in the biosafety cabinet to dry prior to placing the impregnated discs. Impregnated discs containing different dilutions of the extracts, negative control as well as positive control were then placed on the surface of the agar surface, one at a time using a sterile pair of forceps. To facilitate proper infiltration of the extracts into the media, the plates were left for 15 min in sterile environment [20] followed by 37 °C incubation for 24-h [21]. Clear zones of inhibition around the discs were then measured in millimeters (mm) using a ruler and documented in spreadsheets.

### 2.8 Minimum inhibitory concentrations (MICs)

Minimum inhibitory concentration was done in triplicates following the procedure explained by Nikolic et al. [22]. Different concentrations ranging from 100 mg/ml to 1.5625 mg/ml of the extracts were prepared by adding equal volumes (100 μL) of the extracts to Mueller Hinton Broth in different sterile 96-well plates. Thereafter, 20 μL of each bacterial suspension (0.5 McFarland turbidity), was added to each well then incubated for 24-h at 37 °C. This was followed by addition of 50 μL of 1% resazurin solution indicator to each well. The sterile plates were re-incubated for 30 min at 37 °C [23] and the lowest concentration that prevented visible blue to pink resazurin color change was considered the MIC [23]. Similar dilutions were done for Ciprofloxacin (positive antibacterial reference drug) powder, whereas 5% DMSO was utilized as the negative reference.

### 2.9 Minimum bactericidal concentrations (MBCs)

To determine the MBC, 10 μL of the antibacterial agents from every well with concentrations at and above the MIC were streaked over the surface of Mueller Hinton Agar using a sterile cotton swab [18], followed by 37 °C incubation of the agar plates for 24-h. The least concentration with no visible bacterial growth on the Mueller Hinton Agar was considered the MBC [24]. Notable bacterial growth on the surface of Mueller Hinton Agar plates was documented as bacteriostatic effects of the antibacterial agent, whereas lack of visible bacterial growth on the Mueller Hinton Agar plates surface was considered as bactericidal activity of the tested antibacterial agent. Each experiment was done in triplicates.

### 2.10 Quantitative phytochemical screening

Phytochemical screening in the current study was done using GC-MS to determine and quantify phytochemicals available in the ethyl acetate extracts of *X. spekei* and *G. tembensis.* A 7890 A Gas-Chromatograph attached to a 5975C mass selective sensor consisting of an HP5 MS low bleed capillary column was used. The mass spectrometer's operating specifications comprised a relative detector gain mode, a 70eV ionization energy, a 3.3 min' filament delay time, a 1666μ/sec scan speed, a scan range of 40–550 m/z, a 230 °C ion source temperature, as well as a 180 °C quadrupole temperature. A carrier gas of 99.9% helium was used, flowing at a constant rate of 1.25 ml per minute. Mass transfer temperature was set at 200 °C while the injector line transfer temperature set at 250 °C, with an injection volume of 1 μL. Oven temperature was set at 35 °C for 5 min and increased to 280 °C for 24.5 min at a rate of 10 °C per minute. The temperature was then raised to 285 °C for 20.5 min at the rate of 50 °C per minute to a total of 50 min run time.

### 2.11 Data management and statistical analysis

The data in this particular study were tabulated in Microsoft excel spreadsheet before being imported into Minitab software version 17.00, where descriptive statistical values were expressed as mean ± SEM. One-way ANOVA for inferential statistics and Tukey's post hoc test for pairwise comparison and separation of means were used. A p value of <0.05 was considered statistically significant. The findings were presented in tables and graphs. Comparison of the obtained data was matched with mass-spectral library search data from the National Institute of Standards and Technology 08 and 11 to assist in the identification of the phytochemicals found in each extract, where each unique peak represented a particular chemical substance.

## 3 Results

### 3.1 Antibacterial properties

#### 3.1.1 Antibacterial effects of ethyl acetate extracts of *X. spekei* and *G. tembensis* (Fresen)

Antibacterial activities of *G. tembensis* ethyl acetate stem bark extract were tested at different concentrations against bacterial pathogens *S. aureus*, *B. subtilis*, *E. coli*, as well as *S.* Typhi in comparison with the standard antibiotic, Ciprofloxacin, and the diluent, DMSO. The extract exhibited antibacterial activities on *S. aureus* only with MZI ranging from 07.07 mm to 12.33 mm in diameter (Table 1).

**Table 1:** Antibacterial properties of ethyl acetate extracts of *X. spekei* and *G. tembensis* (Fresen).

Treatment	Mean zones of inhibition (mm)			
	*X. spekei*extract		*G. tembensis* stem bark extract	*Grewia tembensis* leaf extract
	*S. aureus*	*B. subtilis*	*S. aureus*	*S. aureus*
5% DMSO	06.00 ± 0.00^f^	06.00 ± 0.00^d^	06.00 ± 0.00^e^	06.00 ± 0.00^e^
Ciprofloxacin (100 μg/ml)	26.33 ± 0.33^a^	29.67 ± 0.33^a^	26.33 ± 0.33^a^	26.33 ± 0.33^a^
Extracts (mg/ml)				
100	14.67 ± 0.33^b^	14.33 ± 0.33^b^	12.33 ± 0.33^b^	11.67 ± 0.33^b^
50	13.33 ± 0.33^b^	13.33 ± 0.33^b^	10.33 ± 0.33^c^	10.00 ± 0.58^bc^
25	11.33 ± 0.33^c^	11.00 ± 0.58^c^	09.33 ± 0.33^c^	08.33 ± 0.33^cd^
12.5	09.67 ± 0.33^cd^	09.67 ± 0.33^c^	07.67 ± 0.33^d^	06.00 ± 0.00^e^
6.25	08.33 ± 0.67^de^	06.00 ± 0.00^d^	07.07 ± 0.07^de^	06.00 ± 0.00^e^
3.125	07.67 ± 0.33^ef^	06.00 ± 0.00^d^	06.00 ± 0.00^e^	06.00 ± 0.00^e^

Values of Mean Zones of Inhibition are expressed as Mean ± SEM. Values with the same superscript letter within the same column are not significantly different (p > 0.05) after one-way ANOVA followed by Tukey's post hoc test.

There was no notable effect against *B. subtilis* (Gram-positive) and Gram-negative (*S****.*** Typhi, and *E. coli*) on all the extract concentrations (Table 1). The ethyl acetate stem bark extract of *G*. *tembensis* at concentration 100 mg/ml, recorded an MZI >12 mm against *S. aureus* which was significantly different from concentrations ranging from 50 mg/ml to 3.125 mg/ml (p < 0.05; Table 1). There was statistical similarity in the antibacterial effects of extract concentrations 50 mg/ml as well as 25 mg/ml on *S. aureus* (p > 0.05; Table 1). Likewise, there was statistical similarity in the effects of extract concentrations 6.25 mg/ml and 12.5 mg/ml and extract concentrations 6.25 mg/ml and 3.125 mg/ml (p > 0.05; Table 1), although there was no activity at concentration 3.125 mg/ml. As the extracts concentrations increased, the inhibition zones against *S. aureus* also increased (Table 1). Ciprofloxacin produced significantly higher inhibitory zones (MZI>25 mm) than all the extract dilutions and DMSO (p < 0.05; Table 1), although DMSO exhibited no activity on all the tested bacterial pathogens (Table 1).

*G. tembensis* leaf extract antibacterial potential was tested at different concentrations against selected bacterial pathogens *S. aureus*, *B. subtilis*, *E. coli*, as well as *S.* Typhi in comparison with the standard antibiotic, Ciprofloxacin, and the diluent, DMSO. The extract exhibited antibacterial activities on *S. aureus* only with MZI ranging from 08.33 mm to 11.67 mm in diameter (Table 1). The extract and DMSO were inactive on *E. coli*, *B. subtilis*, as well as *S.* Typhi in all the extract concentrations (Table 1). Similarly, the extract also showed no activity on *S. aureus* at concentrations ranges of 3.125 mg/ml to 12.5 mg/ml (Table 1). There was statistical similarity in the effect of the extract concentration 100 mg/ml as well as 50 mg/ml (p > 0.05; Table 1). Same statistical similarity was noted in the extract concentration 25 mg/ml and 50 mg/ml (p > 0.05). *G. tembensis* leaf extract's antibacterial effect increased with an increase in concentration (Table 2), with Ciprofloxacin producing significantly larger inhibition zones than all the extract concentrations and DMSO (p < 0.05; Table 1).

**Table 2:** Minimum inhibitory and bactericidal concentrations of ciprofloxacin, *X. spekei* and *G. tembensis* extracts.

	MBC				MIC			
	Concentration (mg/ml)				Concentration (mg/ml)			
Bacterial strain	*X. spekei* extract	*G. tembensis* stem bark extract	*G. tembensis* leaf extract	Ciprofloxacin (μg/ml)	X. spekei extract	*G. tembensis* stem bark extract	*G. tembensis* leaf extract	Ciprofloxacin (μg/ml)
*S. aureus*	33.33 ± 8.33^b^	100.00 ± 0.00^a^	100.00 ± 0.00^a^	1.30 ± 0.26^a^	5.21 ± 1.04^b^	25.00 ± 0.00^a^	33.33 ± 8.33^a^	0.16 ± 0.03^a^
*B*. *subtilis*	100.00 ± 0.00^a^	NA	NA	0.65 ± 0.13^ab^	25.00 ± 0.00^a^	NA	NA	0.16 ± 0.03^a^
*S*. Typhi	NA	NA	NA	0.78 ± 0.00^ab^	NA	NA	NA	0.13 ± 0.03^a^
*E*. *coli*	NA	NA	NA	0.26 ± 0.06^b^	NA	NA	NA	0.05 ± 0.00^a^

Values were conveyed as mean ± std error of mean. Values having similar superscript letters within a particular column are insignificantly distinct after one way Analysis of Variance and Tukey's post hoc (p > 0.05) NA stands for not active.

Ethyl acetate extract of *X. spekei* showed notable antibacterial activities against tested Gram-positive microbes, whereas there was no notable effect against tested Gram-negative microbes (Table 1). *X. spekei* extract showed high effects on *B. subtilis* and *S. aureus* with recorded MZI of >12 mm at extract concentrations between 50 mg/ml and 100 mg/ml (Table 1), although there was statistical similarity in the effects of both extract concentrations (p > 0.05). There was no activity against *B. subtilis* at concentrations 3.125 mg/ml and 6.25 mg/ml. However, *X. spekei* extract showed activity against *S. aureus* in all the tested concentrations (Table 1). Additionally, at 12.5 mg/ml and 25 mg/ml concentrations, there was statistical similarity in antibacterial effects on *B. subtilis* as well as *S. aureus* (p > 0.05; Table 1). Concentrations 6.25 mg/ml and 12.5 mg/ml and concentrations 3.125 mg/ml and 6.25 mg/ml also showed statistical similarities in their antibacterial effects against *S. aureus* (p > 0.05; Table 1). The antibacterial property of *X. spekei* extract on the tested Gram-positive bacteria was concentration-dependent (as concentration increased, extract's activity also increased) (Table 1).

The reference drug, Ciprofloxacin, exhibited a significantly greater effect against all tested microbes than the extract and DMSO (*p* < 0.05; Table 1). The diluent (5% DMSO) demonstrated no activity on all the tested bacterial pathogens (Table 1).

### 3.2 Comparison of antibacterial effects of ethyl acetate extracts of *X. spekei,* and *G. tembensis* against *S. aureus*

**Fig. 1:**
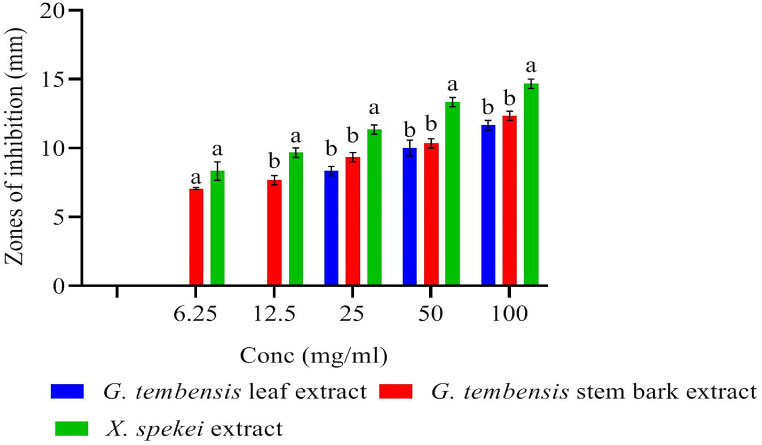
Comparison of antibacterial properties of ethyl acetate extracts of *X. spekei* and *G. tembensis* against *S. aureus*. Bar graphs having distinct letters within a given concentration are significantly insignificant (p < 0.05).

Antibacterial effects of *X. spekei* and *G. tembensis* (leaf and stem bark) extracts were tested against *S. aureus* and compared using one-way ANOVA. *G. tembensis* leaf and stem bark extracts demonstrated statistically similar effects from 25 mg/ml to 100 mg/ml concentrations against *S. aureus* (p > 0.05; Fig. 1). The ethyl acetate extracts of *X. spekei* demonstrated significantly different antibacterial effects from *G. tembensis* leaf and stem bark extracts against *S. aureus,* at 12.5 mg/ml to 100 mg/ml concentration ranges (p < 0.05; Fig. 1). At concentration 6.25 mg/ml, *X. spekei* extract exhibited statistically similar effects with *G. tembensis* stem bark extract against *S. aureus* (p > 0.05; Fig. 1). Comparative activities of *G. tembensis* leaf extract at concentrations range of 3.125 mg/ml to 12.5 mg/ml and *G. tembensis* stem bark extract at concentration 3.125 mg/ml were not done as there were no extract activities at these concentrations (Fig. 1).

### 3.3 Minimum inhibitory concentrations

The test extracts showed varied bacterial growth inhibitions and were thus subjected to minimum inhibitory and bactericidal concentrations depending on the pathogens they had effects on. The mean MIC means ranged from 5.21 ± 1.04 to 25.00 ± 0.00 mg/ml, 25.00 ± 0.00 mg/ml, and 33.33 ± 8.33 mg/ml for ethyl acetate extract of *X. spekei*, stem bark, and leaf extracts of *G. tembensis,* respectively (Table 2). *X. spekei* extract showed significantly different inhibition effects on *B*. *subtilis* as well as *S*. *aureus* (p < 0*.*05; Table 2), while the reference drug, Ciprofloxacin exhibited statistically similar inhibitory effects against all the tested pathogens (p > 0*.*05; Table 2).

In comparison, *X. spekei* extract exhibited inhibitory effects at lower concentrations against *S. aureus* than the other two extracts (Table 2), although its mean inhibitory concentration was statistically similar to that of Ciprofloxacin against *S. aureus* (p > 0*.*05; Table 2). Similarly, the inhibitory potentials of both stem bark and leaf extracts of *G. tembensis* on *S. aureus* had a statistical similarity (p > 0.05; Table 2). *X. spekei* extract's mean inhibitory concentration value was significantly greater than that of Ciprofloxacin on *B. subtilis* (*p <*0*.*05; Table 2).

### 3.4 Minimum bactericidal concentrations

Generally, the tested extracts had significantly greater MBC values than MIC values against each of the tested bacterial pathogens (Table 2). Mean MBC were from 33.33 ± 8.33 to 100.00 ± 0.00 mg/ml, 100.00 ± 0.00 mg/ml and 100.00 ± 0.00 mg/ml for *X. spekei* and *G. tembensis* (stem bark and leaf) extracts respectively (Table 2). *X. spekei* extract showed significantly higher bactericidal effect against *S. aureus* than *B. subtilis* (p < 0.05; Table 2). Additionally, *X. spekei* extract's bactericidal effect against *S. aureus* was at lower concentration than the other two extracts (Table 2). Ciprofloxacin's bactericidal effects were statistically similar against *S. aureus, S.* Typhi as well as *B. subtilis* (p > 0*.*05; Table 2).

In comparison to Ciprofloxacin, the bactericidal effect of *X. spekei* extract against *S. aureus*, was at a significantly higher concentration than Ciprofloxacin (p < 0.05) but at a significantly lower concentration than the other two extracts (p < 0.05; Table 2). Both leaf and stem bark extracts of *G. tembensis* demonstrated statistically similar bactericidal effects against *S. aureus* (p > 0.05). In addition, Ciprofloxacin had bactericidal effects at significantly lower concentrations than all the extracts (p < 0.05; Table 2).

### 3.5 Quantitative phytochemical composition of ethyl acetate extracts of *G. tembensis* stem bark, *G. tembensis* leaf, and *X. spekei*

Phytochemical screening results for stem bark extract of *G. tembensis* as presented in Table 3, showed n-Hexadecanoic acid, a fatty acid derivative, had the highest concentration at 88.06 ± 3.04 μg/g while the compound 2-Pentanone a ketone had the lowest concentration at 0.40 ± 0.00 μg/g. The extract comprised 38.1% fatty acids, 30.6% terpenoids, 11.6% hydrocarbons, 10.2% steroids, 4.0% phenolic compounds, 2.3% aldehyde compounds, 1.4% ketones, 0.6% tocopherol, 0.6% benzene derivatives, 0.4% methoxybenzoic acid compounds and 0.2% aromatic amine compounds.

**Table 3:** Quantitative phytochemical Compounds analysis in ethyl acetate stem bark extract of *G. tembensis*.

Chemical Class	Compound	% abundance	MF	MW (g/mol)	Conc (μg/g)
Ketone	2-Pentanone	0.1	C_5_H_10_O	86.13	0.40 ± 0.00
	5-Decanone	1.3	C_10_H_20_O	156.26	6.93 ± 0.24
Terpenoid	AR- Curcumene	0.3	C_15_H_22_	202.33	1.83 ± 0.07
	2(4H)- Benzofuranone, 5,6,7,7a- tetrahydro- 4,4,7a-trimethyl-	0.4	C_11_H_16_O_2_	180.243	2.03 ± 0.07
	Phytol acetate<E−>	0.6	C_22_H_42_O_2_	338.6	3.02 ± 0.11
	2- Pentadecanone,6,10,14- trimethyl-	0.8	C_18_H_36_O	268.477	4.21 ± 0.15
	Phytol, acetate	0.5	C_22_H_42_O_2_	338.6	2.60 ± 0.09
	4,8,12,16-Tetramethylheptadecan-4-olide	2.6	C_21_H_40_O_2_	324.5	14.05 ± 0.49
	Squalene	2.1	C_30_H_50_	410.7	11.31 ± 0.39
	trans-Geranylgeraniol	0.8	C_20_H_34_O	290.5	4.13 ± 0.14
	2,6,10, 14- Hexadecatetraen-1- ol,3,7,11,15- tetramethyl-, acetate, (E,E,E)-	0.7	C_22_H_36_O_2_	332.5	3.79 ± 0.13
	Ursa- 9(11),12- dien-3-ol	2.4	C_30_H_48_O	424.7	12.97 ± 0.45
	.alpha.-Amyrin	1.4	C_30_H_50_O	426.7	7.81 ± 0.27
	.beta.-Amyrin	0.9	C_30_H_50_O	426.7	4.78 ± 0.17
	Lup-20(29)-en-3-one	3.6	C_30_H_48_O	424.7	19.67 ± 0.68
	Taraxasterol	3.2	C_30_H_50_O	426.7	17.59 ± 0.61
	2,2,4a,6a,8a,9,12 b,14a- Octamethyl- 1,2,3,4,4a,5,6,6a, 6 b,7,8,8a, 9,12,12a,12 b,13,14,14a, 14 b- eicosahydropicene	1.8	C_30_H_50_	410.7	9.77 ± 0.34
	Lupan-3-ol, acetate	3.0	C_32_H_54_O_2_	470.8	16.36 ± 0.57
	Friedelan-3-one	4.3	C_30_H_50_O	426.7	23.17 ± 0.80
Fatty acid and derivatives	Adipic acid, 2-ethylhexyl isobutyl ester	2.5	C_18_H_34_O_4_	314.5	13.76 ± 0.48
	Methyl 18-methylnonadecanoate	1.2	C_21_H_42_O_2_	326.6	6.67 ± 0.23
	Tetracosanoic acid, methyl ester	1.2	C_25_H_50_O_2_	382.7	6.56 ± 0.23
	Heneicosyl acetate	1.4	C_23_H_46_O_2_	354.6	7.42 ± 0.26
	Hexacosyl acetate	1.0	C_28_H_56_O_2_	424.7	5.32 ± 0.18
	Tetracosyl acetate	0.9	C_26_H_52_O_2_	396.7	5.00 ± 0.17
Hydrocarbon	Octacosane	0.3	C_28_H_58_	394.8	1.53 ± 0.05
	Nonadecane	0.3	C_19_H_40_	268.5	1.79 ± 0.06
	Tricosane	1.8	C_23_H_48_	324.6	9.91 ± 0.34
	Tetracosane	1.8	C_24_H_50_	338.7	9.93 ± 0.34
	Pentacosane	1.4	C_25_H_52_	352.7	7.56 ± 0.26
	Hexacosane	1.3	C_26_H_54_	366.7	7.26 ± 0.25
	Tritriacontane	2.7	C_33_H_68_	464.9	14.61 ± 0.50
	Octacosane	1.8	C_28_H_58_	394.8	9.63 ± 0.33
Aldehyde	Tridecanedial	1.4	C_13_H_24_O_2_	212.33	7.85 ± 0.27
	Octadecanal	0.9	C_18_H_36_O	268.5	4.71 ± 0.16
Phenolic compound	Phenol, 2,5-dimethyl-, acetate	2.1	C_10_H_12_O_2_	164.2	11.27 ± 0.39
	Phenol*,* 2*,*4- bis (1*-* methyl-1*-* phenylethyl)-	1.9	C_24_H_26_O	330.5	10.3 ± 0.36
Tocopherol	Vitamin E	0.6	C_29_H_50_O_2_	430.7	3.09 ± 0.11
Phytosterols	Campesterol	3.2	C_28_H_48_O	400.7	17.39 ± 0.60
	Stigmasterol	3.8	C_29_H_48_O	412.7	20.54 ± 0.71
	.gamma.-Sitosterol	1.3	C_29_H_50_O	414.7	7.14 ± 0.25
	Stigmast-4-en-3-one	1.9	C_29_H_48_O	412.7	10.58 ± 0.37
				

**Key: Conc** = Concentration, **Mins** = Minutes, **MF** = Molecular formula, **RT** = Retention time, **MW** = Molecular weight.

The major class of secondary metabolites identified in the leaf extract of *G. tembensis* were 59.33% of terpenoids, 16.17% fatty acids, 15.08% hydrocarbons, 6.58% phenolic compounds, 1.79% ester compounds, 0.79% steroids, 0.19% naphthalene compounds, 0.04% aldehyde, 0.03% toloudine derivative (Table 4). Further, phytochemical investigations of this plant extract showed the presence of 40 compounds with *trans*-3-Penten-2-ol, an alkenol compound, having the lowest concentration at 0.01 ± 0.00 μg/g while Squalene, a triterpenoid, having the highest concentration at 14.17 ± 0.24 μg/g.

**Table 4:** Quantitative phytochemical compounds’ analysis of ethyl acetate leaf extract of *G. tembensis*.

Chemical Class	Compound	%abundance	MF	MW (g/mol)	Conc(μg/g)
Alkenol	*trans*-3-Penten-2-ol	0.02	C_5_H_10_O	86.13	0.01 ± 0.00
Aldehyde	Pivalaldehyde, semicarbazone	0.04	C_6_H_13_N_3_O	143.19	0.03 ± 0.00
Toluidine derivative	Prilocaine	0.03	C_13_H_20_N_2_O	220.31	0.02 ± 0.00
Terpenoids	Limonene	0.22	C_10_H_16_	136.23	0.16 ± 0.00
	2 (4H)- Benzofuranone,5,6,7,7a- tetrahydro- 4,4, 7a-trimethyl-	1.34	C_11_H_16_O_2_	180.24	1.01 ± 0.02
	Bicyclo [3.1.1] heptane, 2,6, 6-trimethyl-, (1.α., 2.β, 5.α.)-	3.92	C_10_H_18_	138.25	2.95 ± 0.05
	2-Pentadecanone, 6,10, 14-trimethyl-	1.14	C_18_H_36_O	268.50	0.86 ± 0.01
	3,7,11, 15-Tetramethyl-2- hexadecen-1-ol	1.22	C_20_H_40_O	296.50	0.92 ± 0.02
	Phytol, acetate	2.06	C_22_H_42_O_2_	338.60	1.55 ± 0.03
	5,9,13- Pentadecatrien-2-one, 6,10,14-trimethyl -,(E,E)-	2.88	C_18_H_30_O	262.40	2.17 ± 0.04
	trans-Geranylgeraniol	2.14	C_20_H_34_O	290.50	1.62 ± 0.03
	Phytol	4.60	C_20_H_40_O	296.50	3.47 ± 0.06
	Phytol acetate<E−>	2.92	C_22_H_42_O_2_	338.60	2.20 ± 0.04
	Squalene	18.78	C_30_H_50_	410.70	14.17 ± 0.2
	.beta.-Amyrin	9.03	C_30_H_50_O	426.70	6.81 ± 0.12
	Lup-20(29)-en-3-one	3.65	C_30_H_48_O	424.70	2.75 ± 0.05
	Taraxasterol	2.31	C_30_H_50_O	426.70	1.75 ± 0.03
	Friedelan-3-one	0.42	C_30_H_50_O	426.70	0.32 ± 0.01
	Nerolidol 1	1.40	C_15_H_26_O	222.37	1.05 ± 0.02
	2,6-Octadienal, 3,7-dimethyl-, (Z)-	1.30	C_10_H_16_O	152.23	0.98 ± 0.02
Fatty acid and derivatives	*Trans*-13- Octadecenoic acid, methyl ester	1.39	C_19_H_36_O_2_	296.50	1.05 ± 0.02
	9,12,15- Octadecatrienoic acid, ethyl ester,(Z,Z, Z)-	5.52	C_20_H_34_O_2_	306.50	4.17 ± 0.07
	Carbonic acid, pentadecyl 2,2,2-trichloroethyl ester	1.14	C_18_H_33_Cl_3_O_3_	403.80	0.86 ± 0.01
	Adipic acid,.beta.-citronellyl tetradecyl ester	0.68	C_28_H_52_O_4_	452.71	0.51 ± 0.01
	13-Tetradecen-1-ol acetate	1.79	C_16_H_30_O_2_	254.41	1.35 ± 0.02
Hydrocarbon	Tricosane	3.10	C_23_H_48_	324.60	2.34 ± 0.04
	Eicosane	1.52	C_20_H_42_	282.50	1.14 ± 0.02
	Tetracosane	3.77	C_24_H_50_	338.70	2.85 ± 0.05
	Octadecane	2.50	C_18_H_38_	254.50	1.89 ± 0.03
	Hexacosane	1.70	C_26_H_54_	366.70	1.28 ± 0.02
Phenolic compound	Phenol, 3,5-dimethyl-	1.58	C_8_H_10_O	122.16	1.19 ± 0.02
	Phenol*,* 2*,*4*-* bis (1*-*methyl*-*1*-* phenylethyl)-	3.34	C_24_H_26_O	330.50	2.52 ± 0.04
	dl-.alpha.-Tocopherol	1.66	C_29_H_50_O_2_	430.70	1.25 ± 0.02
Phytosterols	Stigmasterol	0.16	C_29_H_48_O	412.70	0.12 ± 0.00
Phytosterols	.gamma.-Sitosterol	0.63	C_29_H_50_O	414.70	0.47 ± 0.01

**Key: Conc** = Concentration, **Mins** = Minutes, **MF** = Molecular formula, **MW** = Molecular weight.

Phytochemical compounds of *X. spekei* extract, showed several secondary metabolites in different concentrations with Ursa-9(11),12-dien-3-one, a triterpenoid, having the highest concentration at 38.39 ± 2.40 μg/g whereas 1,16 Cyclocorynan 17-oic acid, 19, 20- didehydro-, methyl ester, (16 S, 19 E)-, an alkaloid, having the lowest concentration at 0.04 ± 0.00 μg/g (Table 5). Additionally, phytochemical screening of ethyl acetate of *X. spekei* extract, revealed 33.34% terpenoids, 27.90% fatty acids, 23.93% steroids, 9.76% hydrocarbons, 1.92% phenolic compounds, 1.02% tocopherols, 0.83% tetracarboxylic acids, 0.97% benzene derivatives, 0.20% ketones, 0.11% dialkylaminodiphenylbutanol ester and 0.02% alkaloid.

**Table 5:** Quantitative analysis of phytochemical compounds in ethyl acetate *X. spekei* extract.

Chemical Class	Compound	%abundance	MF	MW (g/mol)	Conc(μg/g)
Benzene derivative	1-tert-Butyl-3-nitrobenzene	0.37	C_10_H_13_NO_2_	179.22	1.01 ± 0.06
	Benzoic acid, 2, 4-dihydroxy-3, 6-dimethyl-, methyl ester	0.60	C_10_H_12_O_4_	196.19	1.64 ± 0.10
Hydrocarbon	Heneicosane, 10-methyl-	1.09	C_22_H_46_	310.60	2.96 ± 0.19
	1-Docosene	2.53	C_22_H_44_	308.60	6.88 ± 0.43
	Octacosane	0.44	C_28_H58	394.80	1.19 ± 0.07
	Hexadecane	0.96	C16H34	226.44	2.59 ± 0.16
	Hexadecene<1->	0.53	C_16_H_32_	224.42	1.43 ± 0.09
	50.47 Heneicosane	2.27	C_21_H_44_	296.60	6.18 ± 0.39
	Hexacosane	1.71	C_26_H_54_	366.70	4.65 ± 0.29
	Heneicosane	0.23	C_21_H_44_	296.60	0.63 ± 0.04
Dialkylaminodiphenylbutanol ester	Pyrroliphene	0.11	C_23_H_29_NO_2_	351.50	0.29 ± 0.02
Fatty acid and derivatives	Tetradecanoic acid	0.44	C_14_H_28_O_2_	228.37	1.19 ± 0.07
	Methyl stearate	0.49	C_19_H_38_O_2_	298.50	1.33 ± 0.08
	n-Hexadecanoic acid	8.99	C_16_H_32_O_2_	256.42	24.41 ± 1.53
	Pentadecanol<n->	0.57	C_15_H_32_O	228.41	1.56 ± 0.10
	Ethyl hexadecanoate	1.29	C_18_H_36_O_2_	284.50	3.51 ± 0.22
	Linoleic acid ethyl ester	4.68	C_20_H_36_O_2_	308.50	12.7 ± 0.79
	9-Octadecenoic acid, methyl ester, (E)-	0.69	C_19_H_36_O_2_	296.50	1.87 ± 0.12
	Palmitoleic acid	0.37	C_16_H_30_O_2_	254.41	1.00 ± 0.06
	Methyl hexadecanoate	0.27	C_17_H_34_O_2_	270.50	0.73 ± 0.05
Ketone	Z-11-Pentadecenol	0.20	C_15_H_30_O	226.40	0.55 ± 0.03
Phenolic compound	Benzenethiol, 2,4,6-tris(1-methylethyl)-	0.24	C_15_H_24_S	236.40	0.64 ± 0.04
	Phenol, 2,4-bis(1-methyl-1- phenylethyl)-	1.47	C_24_H_26_O	330.50	3.99 ± 0.25
	Phenol, 3,5-bis(1, 1-dimethylethyl)-	0.21	C_14_H_22_O	206.32	0.56 ± 0.03
Alkaloid	1,16- Cyclocorynan −17-oic acid, 19, 20- didehydro-, methyl ester, (16 S, 19 E)-	0.02	C_20_H_22_N_2_O_2_	322.40	0.04 ± 0.00
Terpenoids	4,4, 6a,6 b,8a,11,12, 14 b-Octamethyl-1,4,4a,5,6,6a,6 b,7,8,8a,9,10,11,12,12a,14,14a, 14 b-octadecahydro- 2H-picen-3-one	6.63	C_30_H_48_O	424.70	18.01 ± 1.13
	Squalene	1.46	C_30_H_50_	410.70	3.97 ± 0.25
	Phytol	0.58	C_20_H_40_O	296.50	1.58 ± 0.10
	9-Undecen-2-one, 6,10-dimethyl-	0.95	C_13_H_24_O	196.33	2.59 ± 0.16
	Phytol, acetate	3.52	C_22_H_42_O_2_	338.60	9.57 ± 0.60
	Tributyl acetylcitrate	0.83	C_20_H_34_O_8_	402.50	2.25 ± 0.14
	Ursa-9(11),12-dien-3-one	14.14	C_30_H_46_O	422.70	38.39 ± 2.40
	4,8,12,16-Tetramethylheptadecan-4-olide	2.96	C_21_H_40_O_2_	324.50	8.04 ± 0.50
	Lup-20(29)-en-3-one	1.48	C_30_H_48_O	424.70	4.03 ± 0.25
	Widdrol	0.49	C_15_H_26_O	222.37	1.34 ± 0.08
	Lanost-8-en-3-one	0.82	C_30_H_50_O	426.70	2.21 ± 0.14
	2-Pentadecanone, 6,10,14-trimethyl-	0.31	C_18_H_36_O	268.50	0.84 ± 0.05
Tocopherol	2H-1-Benzopyran-6-ol, 3,4-dihydro- 2,8-dimethyl-2- (4,8,12 -trimethyltridecyl)-, [2 R-[2 R*(4R*,8R*)]]-	1.02	C_27_H_46_O_2_	402.65	2.76 ± 0.17
Phytosterols	9,19- Cyclo-25, 26-epoxyergostan-3-ol, 4,4, 14- trimethyl-, acetate	1.21	C_33_H_54_O_3_	498.78	3.28 ± 0.21
	Stigmast-4-en-3-one	5.96	C_29_H_48_O	412.70	16.19 ± 1.01
	17- (1,5-Dimethylhexyl)-10,13- dimethyl-2,3,4,7,8,9,10,11, 12,13,14,15,16, 17-tetradecahydro-1h-cyclopenta[*a*]Phenanthren-3-ol	2.18	C_27_H_48_O	386.65	5.93 ± 0.37
	Stigmasterol	0.87	C_29_H_48_O	412.70	2.36 ± 0.15
	4,22-Stigmastadiene-3-one	1.02	C_29_H_46_O	410.70	2.78 ± 0.17
	.gamma.-Sitosterol	12.69	C_29_H_50_O	414.70	34.46 ± 2.15

**Key: Conc** = Concentration, **Mins** = Minutes, **MF** = Molecular formula, **RT** = Retention time, **MW** = Molecular weight.

## 4 Discussion

Herbal plants are known to produce secondary metabolites with known effects against bacterial pathogens and less adverse effects in comparison to conventional antibacterial agents [25]. Various plants have been used traditionally as antibacterial agents however, they lack scientific validation and documentation on their usage. This current study determined the *in vitro* antibacterial effects of the ethyl acetate extracts of *X. spekei* and *G. tembensis* stem bark and leaves against *E. coli*, *B. subtilis*, *S.* Typhi, as well as *S. aureus*.

This is considerably the initial report about the antibacterial effects of ethyl acetate stem bark extracts of *G. tembensis*. However, previous studies have shown antibacterial capabilities of stem bark extracts of other plants of the genus *Grewia*. For instance, the ethanol stem bark extracts of *Grewia mollis* have exhibited antibacterial potentials on *E. coli, S. aureus* as well as *Streptococcus* sp [26]. The ethyl acetate stem bark extracts of *G. tembensis* exhibited notable antibacterial effects against *S. aureus* only with MZI ranges of 07.07 ± 0.07 to 12.33 ± 0.33 mm. This concurs with a report by Akwu et al. [27], which confirmed that lupeol compounds from the stem bark of *Grewia lasiocarpa* were inactive on *S.* Typhi and *E. coli*.

Similarly, this is considerably the initial study about the antibacterial potential of ethyl acetate leaf extracts of *G. tembensis*. However, previous experiments have demonstrated that the leaves of other *Grewia* genus plants have antibacterial activities. The methanol, n-hexane, and ethyl acetate leaf extracts of *Grewia pubescens* have been shown to possess antibacterial properties [28]. Ethyl acetate leaf extracts of *G. tembensis* exhibited antibacterial capability against only *S. aureus* in a dilution-dependent trend with MZI ranging from 08.33 ± 0.33 to 11.67 ± 0.33 mm. These findings agrees with a study which indicated that the ethyl acetate leaf extracts of *G. plagiophylla* displayed no antibacterial potential against *E. coli* and *S.* Typhi but had activity against *S. aureus* [29]. This was also in agreement with a study that found no activity against *B. subtilis* when the methanol and ethanol fruit extracts of *Phoenix dactylifera* and ethanol and methanol seed extracts of *Clitoria ternatea* were tested on *B. subtilis, S.* Typhi*, B. cereus*, and *E. coli* [30].

The current study noted that there is lack of published report available on antibacterial capabilities of ethyl acetate extracts of *X. spekei*. *Xerophyta retinervis* which belongs to the same genus as *X. spekei* has illustrated antimicrobial, antiulcer as well as antiinflammatory potentials. In South Africa, *X. retinervis* is used by traditional herbalists to cure rhinitis and headache [31]. The whole plant of *X. retinervis* is traditionally used in the Pretoria region of South Africa as a therapy for asthma and nose bleeding [32]. The ethyl acetate extract of *X. spekei* produced notable inhibition zones beginning from 07.67 ± 0.33 to 14.67 ± 0.33 mm against *S. aureus* and inhibition zones beginning from 09.67 ± 0.33 to 14.33 ± 0.33 mm against *B. subtilis* but had no antibacterial activities on the tested Gram-negative microbes. This was in consensus with Sapkota et al. [33], who showed that ethanol leaf extracts of *Artimisia vulgaris* and *Eupharbia hirta* had an antibacterial impact on *B. subtilis* and *S. aureus* but were inactive on *K. pneumoniae*, *E*. *coli*, *S. dysenteriae,* as well as *S*. Typhi. Additionally, this also agrees with the findings of a study by Seaman [34] that illustrated that ethyl acetate extracts of *X. retinervis* had antibacterial activity on *S. aureus*.

The antibacterial effects of the tested plant extracts in the current study are attributed to the presence of various secondary metabolites. Compounds like terpenoids have been known to exhibit antibacterial effects by interfering with bacterial oxygen uptake and oxidative phosphorylation which are two important essential processes in microorganisms [5]. Phytol, a diterpenoid which was found in all the studied extracts, has known antibacterial activity [35]. Additionally, Lup-20(29)-en-3-one, a triterpenoid, which was also present in *X. spekei* and *G. tembensis* extracts, has also been known to have antibacterial effects [36].

Alkanes act by interfering with bacterial cell membrane integrity causing bacterial cell death [37]. Octacosane, a straight-chain alkane, which was found in *G. tembensis* stem bark and *X. spekei* extracts, has known antibacterial properties [38]. Another hydrocarbon, Heneicosane, which was found in both *X. spekei* and *G. tembensis* leaf extracts, has previously shown antibacterial activities [39].

Fatty acids act by inhibiting bacterial enzyme activity, and direct bacterial cell lysis [40]. Fatty acid like n-Hexadecanoic acid (palmitic acid), found in all the tested extracts, has been shown to possess antimicrobial effects [41]. Another fatty acid, Tetradecanoic acid, that was present in both *X. spekei* and *G. tembensis* stem bark extracts, has previously been shown to have antibacterial effects [42].

Phenolic compounds, are known to cause bacterial cell lysis in addition to membrane-disturbing properties as their mode of antibacterial efficacy [43]. Phenol, 3,5-bis (1, 1-dimethylethyl)-, which was present in *X. spekei* extract has been shown to have antibacterial activities [44]. Phenol, 2,5-dimethyl-, acetate which was found in *G. tembensis* stem bark extract has displayed antimicrobial activities [45].

Stigmasterol, a phytosterol which was present in *X. spekei, G. tembensis* stem bark, and leaf extracts, has been previously shown to have antibacterial activities [46]. Stigmast-4-en-3-one, also a phytosterol, present in both *X. spekei* and *G. tembensis* stem bark extracts, has been shown to have antibacterial effects [47].

This study had some limitations. The first limitation was lack of previous published data which led to limited access to information on antibacterial activities of the studied plant extracts leading to limited reference and comparison to the activity of these studied plants. The second limitation was limited availability of resources which limited our capability in conducting more biological assays.

## 5 Conclusions

The current experiment aimed at confirming the traditional use of *X. spekei* and *G. tembensis* medicinal plants against *E. coli, B. subtilis, S.* Typhi, and *S. aureus* by investigating their *in vitro* antibacterial potential and the presence of phytochemicals associated with antibacterial properties. The ethyl acetate extracts of the studied plant extracts demonstrated varied antibacterial potentials with *X. spekei* extract, exhibiting activity on *S. aureus* and *B. subtilis* only while both the stem bark and leaf extracts of *G. tembensis* exhibited activity on *S. aureus* alone. Overall, our current findings indicate that the studied extracts can be potential candidates to extract therapeutic antibacterial agents for managing and treating bacterial illnesses.

## Author contribution statement

Paul Ochieng Nyalo: Conceived and designed the experiments; Performed the experiments; Analyzed and interpreted the data; Contributed reagents, materials, analysis tools or data; Wrote the paper. George Isanda Omwenga, Mathew Piero Ngugi: Conceived and designed the experiments; Contributed reagents, materials, analysis tools or data.

## Funding statement

This research did not receive any specific grant from funding agencies in the public, commercial, or not-for-profit sectors.

## Data availability statement

Data included in article/supplementary material/referenced in article.

## Ethical approval

The experimental procedures and protocols in this study were approved by Kenyatta University Graduate School approval committee.

## Declaration of competing interest

The authors declare no conflict of interest.
